# BK viruria and viremia in children with systemic lupus erythematosus

**DOI:** 10.1186/s12969-017-0156-2

**Published:** 2017-04-11

**Authors:** Nirupama Gupta, Cuong Q. Nguyen, Renee F. Modica, Melissa E. Elder, Eduardo H. Garin

**Affiliations:** 1 Division of Nephrology, Department of Pediatrics, College of Medicine, University of Florida, Gainesville, FL 32610 USA; 2 Department of Infectious Diseases and Pathology, College of Veterinary Medicine, University of Florida, Gainesville, FL 32610 USA; 3 Division of Immunology, Rheumatology and Infectious Diseases, Department of Pediatrics, University of Florida, Gainesville, FL USA

**Keywords:** SLE, Pediatric, Biologics, BK virus, Infections, Immunosuppression

## Abstract

**Background:**

BK virus (BKV) is a ubiquitous polyoma virus that lies dormant in the genitourinary tract once acquired in early childhood. In states of cellular immunodeficiency, the virus can reactivate to cause hemorrhagic cystitis and nephritis. Children with systemic lupus erythematosus (SLE) have an increased risk of developing infectious complications secondary to their immunocompromised state from the administration of several immuno-modulatory drugs. Currently, there are no data regarding the prevalence of BK viruria or viremia in children with SLE.

**Methods:**

We conducted a prospective cohort study involving children with SLE of 18 years and younger. We obtained urine and blood samples at baseline and every 3 months up to 1 year for BK virus detection by real-time, quantitative polymerase chain reaction analysis. A comprehensive review of demographic information, clinical characteristics and medication history was also obtained.

**Results:**

Thirty-two pediatric patients (26 females and 6 males) with SLE were enrolled. Median age at the time of SLE diagnosis and enrollment into study was 13.6 years and 16.0 years old, respectively. The prevalence at enrollment was 3.1% (1/32) for BK viruria and 6.2% (2/32) for BK viremia. During the study period, 3 patients had viruria, 5 had viremia and 4 had both viruria and viremia. Of the 12 patients with BKV reactivation, only one was positive for microscopic hematuria, all others were asymptomatic. A total of nine of 97(9.2%) urine samples and 10 of 96(10.4%) blood samples were positive for BK virus. The most commonly utilized biologics in this cohort group were Rituximab (90.6%), Abatacept (12.5%), and Belimumab (9.3%). The type of medication exposure and clinical characteristics did not statistically differ between the groups that did or did not have BK viruria and/or viremia.

**Conclusions:**

Our study suggests that pediatric patients with SLE have BK viremia and/or viruria at a higher rate than the general healthy population, although the significance of the reactivation and viral level is unclear. The influence of immune-modulatory drugs on BKV reactivation is still uncertain. To understand the interplay amongst BK virus, immunosuppression and dysregulated immune system in children with SLE, ongoing research in a larger population is still warranted, which may help establish proper surveillance, diagnosis and treatment for BKV infection.

## Background

BK virus (BKV) was first isolated in 1971 from the urine of a renal transplant patient with ureteric stenosis. The BKV is a small (~45 nm) icosahedral non-enveloped double stranded DNA virus. It consists of three domains that contain the replicative genes (T antigen), transcription factors, and the viral capsid proteins (VP1, VP2, VP3) [1, 2]. It is acquired during early childhood and lies dormant in the genitourinary tract, often localized to the renal medulla [3]. It is known to be associated with hemorrhagic cystitis and nephropathy leading to renal failure.

The microenvironment needed for BKV replication includes an interplay between the viral characteristics, host’s altered or impaired immune system, inflammation and/or intrinsic kidney damage [4]. It is known that asymptomatic viruria occurs in both healthy and immunocompromised patients, with occurrence of < 5% in the healthy population and up to 60% in immunocompromised patients (both solid-organ transplant and non-transplant recipients) [5, 6]. Other sources site that about 21.5% and 6.7% of healthy adults and children, respectively, have asymptomatic BK viruria [7]. Children with autoimmune disorders such as systemic lupus erythematosus (SLE) tend to have a higher degree of renal involvement (nephritis, acute kidney injury, nephrotic syndrome) in addition to an impaired/dysregulated immune system, which may possibly place them at a higher risk for BK virus replication than adults [8].

Typically, an intact cellular immune response is required to clear viral infections. The use of immunosuppressive drugs, including biologics, impacts several components of the host’s immune system including humoral, cellular and/or cytokine signaling. An altered immune system can therefore increase the risk of opportunistic bacterial, viral, and fungal infections [9]. It is reported that patients exposed to monoclonal antibodies such as Natalizumab [10], Infliximab and Adalimumab [11], and Rituximab [12] had a prevalence of BK viruria of 22%, 55%, and 63% respectively. The prevalence rate at baseline before starting Natalizumab and Rituximab was 8.3% [10] and 27% [12], respectively. Therefore, an impeded cellular immune response to elevated BK viral loads might contribute to the occurrence of higher rates of BK viruria and/or viremia in children with SLE [13, 14].

Currently, there is a lack of clinical data regarding the association between the prevalence of BK viruria and viremia in children with SLE and the impact of immunosuppressive drugs. This is the first study that examines the relationship between BKV and children with SLE. With enhanced understanding of the interplay amongst BKV, immunosuppression and dysregulated immune system in children with SLE, we may be able to establish proper surveillance, diagnosis and treatment for BKV infection.

## Methods

### Study population

This prospective cohort study was performed at the Division of Pediatric Immunology and Rheumatology at the University of Florida in Gainesville from January 2014 to February 2016. Study enrollment included children ≥ 4yo and ≤18 years old with SLE who presented to the pediatric rheumatology outpatient clinic and inpatient rheumatology service. We included both former and new diagnoses of SLE based on the 1997 American College of Rheumatology criteria. Subjects with history of mixed connective tissue disease (MCTD) and/or overlap syndrome were not excluded if they met the criteria for SLE at the time of enrollment. Subjects with a solid organ and/or bone marrow transplant were excluded. Subjects with cancer, on dialysis, or with human immunodeficiency virus (HIV) infection were also excluded in this study. The protocol was approved by the University of Florida Institutional Review Board (IRB201400023). Informed consent/assent was obtained from all subjects and guardian.

### Specimen collection and handling

Urine and blood samples from all subjects (former or new SLE diagnoses) were obtained at study entry and at follow-up visits at 3, 6 months and 9–12 months. A clean catch urine sample was collected in a sterile container and 3–5 ml of blood was collected in single EDTA tubes (BD, catalog number 367835).

### Chart Abstraction

A comprehensive medical record review for demographic information, baseline clinical and laboratory characteristics of the disease and immunosuppressive and immunomodulatory treatments administered was completed (see Table 1).

**Table 1:** Baseline clinical and laboratory characteristics

Clinical findings	At time of enrollment (*N* =32)
Female (%)	26 (81.3%)
Age (years)	16.0
Weight (kg)	61.5 +/- 20.35
Ethnicity (%)	
African American	9 (28%)
Caucasian	12 (37.5%)
Hispanic/Other	11 (34.3%)
Age at diagnosis of SLE (years)	13.67
Age at enrollment (years)	16.0
Duration of SLE (Months)	22.9 +/- 20.51
New onset SLE (%)	4 (12.5)
Lupus nephritis WHO* (%)	
I	4 (12.5)
II	2 (6.3)
III	1 (3.1)
III/V	1 (3.1)
IV	11 (34.4)
V	4 (12.5)
No renal biopsy	9 (28.1)
H/O antiphospholipid syndrome	1
H/O neuropsychiatric manifestation/CNS Lupus	6
H/O Vasculitis	4
H/O Arthritis	25
H/O Serositis	3
H/O Pulmonary hemorrhage	1
H/O Cytopenia	22
H/O Raynaud’s phenomeonoN	3
H/O Positive anti-double stranded DNA	22
Positive ANA	32
Ribonuceloprotein	21
Laboratory findings	At time of enrollment [mean]
Hemoglobin (g/dl)	12.40
White blood counT (X 10^9^/L)	5.75
Platelet count (X 10^9^/L)	255
Serum creatinine (mg/dl)	0.64
Complement 3 (g/dl)	110
Complement 4 (g/dl)	19
CD3	1004
CD19	0
IGG	820
IGA	128
IGM	29
Urinalysis	
Hematuria (%)	5 (15.6)
Proteinuria (%)	6 (18.75)
Normal (%)	21 (65.6)

*WHO—World Health Organization

### Extraction and Purification of DNA from Plasma and Urine

The plasma fraction was extracted from the blood by low-speed centrifugation. About 200ul of plasma per replicate was used to isolate total DNA. The QIAamp DNA Blood Mini kit (Qiagen) was used to isolate DNA via the manufacturer’s instruction. Similar procedure was utilized for urine BK viral DNA isolation. Samples were frozen at −80 °C after collection and assayed as a batch on a later date.

### Real-time polymerase chain reaction (RT-PCR)

RT-PCR was used to detect and quantify BK virus [15]. Primers were designed to amplify the capsized protein (VP-1) of BKV with forward primer: 5’ GCA GCT CCC AAA AAG CCA AA 3’ and reverse primer, 5’ CTG GGT TTA GGA AGC ATT CTA 3’. The BKV Dunlop strain plasmid obtained from American Type Culture Collection was used as positive control. PCR amplifications were performed using iTaq Universal SybrGreen Supermix (Bio-Rad) with the following PCR conditions: Thermal cycling was initiated with a denaturation step at 95 °C for 10 min, followed by 40 cycles of 95 °C for 10 s and 60 °C for 10 s.

Quantitative real-time PCR assays were performed using Bio-Rad CFX96 Realtime PCR detection system. RT- PCR amplification data was analyzed with the software provided by the manufacturer (ABI 7500). Samples were run as duplicates. Data was expressed as copies of viral DNA per milliliter of urine or plasma. Negative control lanes for each run were also included. BK viruria and BK viremia was defined as the presence of BK DNA PCR greater than zero copies/ml in the urine and blood, respectively.

### Statistics

Statistics were performed using Graph Prism Pad. Parametric statistical tools were utilized. Fisher’s exact test was used to assess the risk factors associated with presence or absence of BK viremia/viruria. The prevalence was the fraction of subjects with BK viremia/viruria at baseline visit and at the 12 month follow-up. The incidence rate was restricted to the subjects negative at baseline.

## Results

A. Patients and Laboratory Findings (Table 1)
  Thirty-two pediatric patients (26 females and 6 males) with SLE were enrolled, of which 81% were females. Equal representation of three ethnicity groups (African American, Caucasian, and Hispanic) were present. MCTD and overlap syndrome were also diagnosed in 7 (22%) and 3 (9%) of 32 patients, respectively. Median age at the time of SLE diagnosis and enrollment into study was 13.6 years and 16.0 years old, respectively. Four new patients (12.5%) were enrolled at the time of SLE diagnosis. Median duration of SLE was about 22.9 months since diagnosis to time of enrollment. Seventy-two percent had biopsy proven SLE nephritis, while 28% did not have a biopsy done. At the time of enrollment, abnormal urinalysis was present in 32% of patients and the median serum creatinine was 0.64 mg/dL. None of the patients had presented with acute renal failure.

B. BK Viruria and/or BK Viremia
  Of the 32 enrolled patients, 13 (40.6%) completed the 12-month study period, while others were lost to either follow-up or missed scheduled clinic appointments (Table 2). BKV reactivation and quantity varied temporally, and that viremia occurred without viruria at times. Nine of 97 urine samples (9.2%) and 10 of 96 blood samples (10.4%) were positive for BKV. Twelve of 32 individuals (a 12 month period prevalence rate of 37.5%) had either BKV positive in the urine or blood during this study period, of which 6 (50%) also had history of MCTD and/or overlap syndrome. The prevalence at enrollment was 3.1% (1/32) for BK viruria and 6.2% (2/32) for BK viremia. The highest prevalence of BK viruria (18%, 4 of 22) was at 3 months into the study. The highest prevalence of BK viremia (31.5%, 6 of 19) was at 6 months into the study. Only 3 of 12 (25%) patients had multiple positive urine or blood samples for BKV at different time intervals. The incidence for new onset BK viruria and viremia in patients was 18.8% (6 of 32) and 21.8% (7 of 32), respectively. The number of BK virus copies/ml in the urine and blood ranged from 0.03–1.86 ×10^4^ and 0.68–1.5x 10^6^, respectively. Seventeen of 97 urine samples had proteinuria [7] or hematuria [10] on urinalysis. Of these samples, only one patient with microscopic hematuria had concurrent positive BKV viremia without viruria (#27, at baseline). Besides the previous patient (#27), all other 11 patients identified with BK viruria and/or viremia were asymptomatic (negative for proteinuria and hematuria on urinalysis).

**Table 2:** BK viruria and/or viremia in all enrolled patients ^a^

Patient	At enrollment		3 months		6 months		9 months		12 months	
	Urine	Blood	Urine	Blood	Urine	Blood	Urine	Blood	Urine	Blood
1	Negative	Negative	Negative	Negative	Negative	***Positive*** ***15.6***	Negative	Negative	n/a	n/a
2	Negative	Negative	Negative	Negative	Negative	Negative	n/a	Negative	n/a	Negative
3	Negative	Negative	Negative	Negative	n/a	***Positive*** ***30.9***	Negative	Negative	Negative	Negative
4	Negative	Negative	Negative	Negative	***Positive*** ***0.03***	***Positive*** ***2.65***	Negative	Negative	Negative	Negative
^b^ 5	Negative	Negative	Negative	Negative	***Positive*** ***1.04×10*** ^***4***^	***Positive*** ***0.68***	***Positive*** ***1.86×10*** ^***4***^	Negative	***Positive*** ***1.76×10*** ^***3***^	Negative
6	Negative	Negative	***Positive*** ***527***	Negative	Negative	***Positive*** ***4.5***	n/a	n/a	Negative	Negative
7	Negative	Negative	n/a	n/a	n/a	n/a	n/a	n/a	n/a	n/a
8	Negative	Negative	***Positive*** ***1.60***	Negative	Negative	Negative	n/a	n/a	Negative	Negative
^b^ 9	Negative	***Positive*** ***1.5x10*** ^***6***^	Negative	Negative	Negative	***Positive*** ***4.3***	Negative	Negative	Negative	Negative
10	Negative	Negative	n/a	n/a	n/a	n/a	n/a	n/a	n/a	n/a
11	Negative	Negative	n/a	n/a	n/a	n/a	n/a	n/a	n/a	n/a
^b^ 12	Negative	Negative	n/a	Negative	n/a	n/a	n/a	n/a	n/a	n/a
^b^ 13	Negative	Negative	***Positive*** ***563***	n/a	Negative	Negative	n/a	n/a	n/a	n/a
^b^ 14	Negative	Negative	Negative	Negative	Negative	Negative	n/a	n/a	n/a	n/a
^c^ 15	Negative	Negative	n/a	n/a	n/a	n/a	Negative	Negative	n/a	n/a
16	Negative	Negative	n/a	n/a	n/a	n/a	n/a	n/a	Negative	Negative
17	Negative	Negative	n/a	n/a	n/a	n/a	n/a	n/a	n/a	n/a
18	Negative	Negative	n/a	n/a	n/a	n/a	n/a	n/a	n/a	n/a
^b^ 19	Negative	Negative	Negative	Negative	Negative	Negative	n/a	n/a	Negative	Negative
20	Negative	Negative	Negative	n/a	Negative	Negative	Negative	n/a	n/a	n/a
21	Negative	Negative	n/a	n/a	Negative	Negative	n/a	n/a	n/a	n/a
22	Negative	Negative	Negative	***Positive*** ***26.5***	Negative	Negative	Negative	Negative	n/a	n/a
^b^ 23	Negative	Negative	***Positive*** ***2.47***	***Positive*** ***2.84***	n/a	n/a	n/a	n/a	Negative	Negative
24	Negative	Negative	Negative	Negative	Negative	Negative	n/a	n/a	n/a	n/a
25	Negative	Negative	Negative	Negative	Negative	Negative	Negative	Negative	Negative	Negative
^c^ 26	***Positive*** ***0.07***	Negative	Negative	Negative	Negative	Negative	Negative	Negative	Negative	Negative
^c^ 27	Negative	***Positive*** ***33.5***	Negative	n/a	n/a	n/a	n/a	n/a	n/a	n/a
28	Negative	Negative	Negative	Negative	Negative	Negative	n/a	n/a	n/a	n/a
29	Negative	Negative	n/a	n/a	n/a	n/a	Negative	Negative	n/a	n/a
30	Negative	Negative	Negative	Negative	Negative	Negative	Negative	Negative	Negative	Negative
31	Negative	Negative	Negative	n/a	n/a	n/a	Negative	Negative	n/a	n/a
32	Negative	Negative	Negative	Negative	n/a	n/a	n/a	n/a	n/a	n/a
Positive BKV/Total Samples (%)	1/32 (3.1%)	2/32 (6.2%)	4/22 (18.1%)	2/19 (10.5%)	2/18 (11.1%)	6/19 (31.5%)	1/13 (7.6%)	0/13 (0%)	1/12 (8.3%)	0/13 (0%)

^a^ BKV copies/ml

^b^ MCTD

^c^ Overlap syndrome, n/a—not available

Bold italic indicates positive tests
  The only identifiable difference noted between the two groups with or without BK viremia and/or viruria was exposure to hydroxychloroquine (Table 3). Age, gender, ethnicity, SLE class, hematuria, proteinuria, cytopenia, serum creatinine level, complement C3 and C4 levels, immunoglobulin level and the exposure to biologics were not statistically significant between the group that did or did not develop BK viremia and/or viruria.

**Table 3:** Associated Risk Factors for the presence of BK Viruria and/or Viremia in children with SLE at the time of enrollment

Clinical history	Presence of Bk viruria or viremia (*N* = 12)	Absence of BK viruria or viremia (*N* = 20)	*P*-value
	Median	Median	
Age	15	16	0.08
Female	9	17	0.65
Male	3	3	
Race			0.79
Caucasian	4	8	
African American	3	6	
Other	5	6	
SLE WHO Class			0.75
I	2	2	
II	1	1	
III	0	1	
III/IV	1	0	
IV	3	8	
V	2	2	
No renal biopsy	3	6	
Duration of SLE	21	21	0.89
Laboratory findings			
Serum creatinine	0.61	0.66	0.33
Hematuria	1	2	1.0
Proteinuria	2	4	1.0
Hemoglobin	12.45	12.25	0.56
Platelet count	269.5	243	0.77
WBC Count	5.05	5.95	0.89
C3	106	113	0.77
C4	18	19	0.88
CD3	982	1087	0.44
CD19	0	0	0.23
IGG	796	866	0.80
IGA	86	143	0.21
IGM	30	29	0.88
Medication history			
Exposure to any biologic ^a^	11	16	0.62
1 Biologic	10	13	
2 Biologics	0	2	
3 OR more biologics	1	1	
Exposure to IVIG	5	6	0.70
Exposure to hydroxychloroquine	12	9	0.0016 ^b^
Exposure to MMF	9	17	0.65
Exposure to cyclophosphamide	3	11	0.15
Exposure to rituximab	10	15	0.68
Exposure to steroids	12	20	1.0

*N* number of patients

^a^ Two biologics (Rituximab and Tocilizumab) were given simultaneously in only one patient (patient # 5)

^b^ Statistically significant *p*-value

C. Medication Exposure
  The most commonly utilized biologics (Table 4) in this cohort group were Rituximab (90.6%), Abatacept (12.5%), and Belimumab (9.3%), with a median of 8, 21, 8 doses given respectively. The median exposure time, the time from initiation of drug to either time of discontinuation or end of study period, for Rituximab, was 503 days (range 96–1226 days). Twelve patients received IVIG, with exposure time of 241.5 days (range 1–1462 days). The most commonly non-biologics utilized were Cyclophosphamide (46.8%), Hydroxychloroquine (78.1%), Mycophenolate mofetil (MMF) (87.5%), Prednisone (93.7%) and Solu-medrol (100%). The median exposure time for Cyclophosphamide, MMF, prednisone and Solumedrol were 148, 921.5, 525.5, and 547.5 days, respectively.

**Table 4:** Medication exposure from the time of drug initiation to the end of study period

Type of biologic exposure	No. of patients [*N* = 32 (%)]	Overall cumulative dose (grams) [median, range]	Cumulative dose/body weight (mg/kg) [median, range]	# of doses [median, range]	Length of exposure since diagnosis in days [median, range]
Abatacept	4 (12.5)	15.3 (3.6–26.2)	231.6 (108–375)	21 (8–28)	540.5 (169–732)
Adalimumab	1 (3.1)	480	10.2	12	159
Belimumab	3 (9.3)	3.9 (1–5.3)	75.5 (18.7–78.1)	8 (2–8)	176 (16–235)
Infliximab	2 (6.25)	4.7	91.8	11.5	299
Rituximab	29 (90.6)	5.0 (1.8–9.9)	89.1 (18–192.1)	8 (2–12)	503 (96-12,226)
Tocilizumab	2 (6.25)	3.4	57.9	8	196.5
Ivig (g/kg)	12 (37.5)	263.5 (1–14,193)	6.85 (1–39.8)	11.5 (1–47)	241.5 (1-1462)
Ever received Non-biologics	No. Of patients [*N* = 32 (%)]	Overall cumulative dose (grams) [median, range]	Cumulative dose/body weight (mg/kg) [median, range]	# of doses [median, range]	Length of exposure since diagnosis in days [median, range]
Azathioprine	2	37.7	790.6	N/A	496
Cyclophosphamide	15 (46.8)	7.1 (4.3–12.7)	94.6 (40.3–295)	6	148 (84-1152)
Hydroxy-chloroquine	25 (78.1)	N/A	N/A	N/A	536 (72–2801)
Methotrexate	4 (12.5)	1.2 (0.5–2.3)	20.3 (11.9–42.7)	N/A	354 (224–1167)
Mycophenolate	28 (87.5)	1109 (128–6751)	23,680 (1950–400,720)	N/A	921.5 (38-2261)
Prednisone	30 (93.7)	6.1 (0.1–25.4)	95.35 (2.3–542.4)	N/A	525.5 (5-3987)
Solu-medrol	32 (100)	10.4 (3–60)	158.9 (41–1272)	16 (3–60)	547.5 (1-1788)

*N/A* not applicable
  The type of medication exposure did not statistically differ between the groups, except for hydroxychloroquine, that had presence of BK viruria and/or viremia (Table 3). In both groups, Rituximab, Solumedrol, Cyclophosphamide, and MMF were the most frequent drugs given.

## Discussion

Taguchi et al. [16] first reported the presence of BK virus in SLE in 1979. Since then the prevalence of BKV viruria in adult patients with SLE has been reported to be 16% and about 26% of those have persistent or recurrent BK viruria after 1 year of follow-up [17]. In our study, we prospectively followed a cohort of children with SLE for 1 year. We also observed that only 28% (9 of 32) had BK viruria and 22% (7 of 32) had BK viremia, as defined by the presence of BKV on PCR greater than zero copies/ml. Our findings cannot be directly compared with other studies on BKV in SLE due to use of different arbitrary viral cut-off levels, which can under- or overestimate the true prevalence. Colla et al [18] used a cut off of >1000 BKV copies/ml and Lu et al [19] used positive BK viruria as >50,000 BKV copies/ml in adults with SLE. Moreover, they tested on only single urine samples. However, in agreement with other studies like Sunjsford [11] and Flores [8], we also show that SLE patients have waxing and waning of BKV positivity at different times over a 12-month period. .

We also observed a wide range of BK viral load in the urine and blood, even in the same patient. We also noticed that BK viruria did not always precede BK viremia. This is contrary to Nickeleit et al [8] who reported that viruria often precedes viremia by several weeks. This finding highlights the importance of checking both the urine and blood for BK virus infection and raises the question of BK virus tissue tropism. The BK viruria and/or viremia was transient and and the clinical findings of proteinuria, hematuria or elevated serum creatinine levels did not strongly support infection, which is similar to findings noted by Colla et al [18], Lu et al [19], and Rainthavorn et al [20]. We did not find a relationship between the incidence of thrombocytopenia and BK virus as reported by Lu et al [13].

The current era of immunosuppression has begun to utilize more biologics and children who have SLE are exposed to such agents at a younger age. Almost everyone in our cohort received Rituximab (anti-CD20 monoclonal antibody), and a smaller percentage had received Belimumab (anti B lymphocyte stimulator), Abatacept (anti-CTLA4 Ig), Infliximab (anti-TNF-α), and Tocilizumab (anti-IL6 receptor). In this cohort Infliximab, Adalimumab and Tocilizumab were used in patients who met criteria for SLE in the context of either MCTD or Overlap syndrome, and were used to treat non-lupus features. Of interest, of the pediatric SLE patients with a positive BK viruria or viremia, about half also had diagnosis of MCTD and/or overlap syndrome with non-lupus features such as erosive and persistent arthritis, dermatomyositis and/or vasculitis. It is unclear what this finding signifies at this time and whether or not their underlying immune dysregulation or specific immunosuppressive agents (Infliximab or Tocilizumab) used for these conditions differs with regard to BKV risk. We did not find that steroids or cyclophosphamide exposure, which is associated with higher risk for infection [21], influenced the presence of BK virus. It is unclear why the antimalarial hydroxychloroquine in this study was associated with a higher rate of BK virus presence (*p* = 0.016), when they are supposed to have a protective role [22]. The results may be influenced by the small sample size. In our study, IVIG was used in 37.5% (12 of 32) patients for the treatment of secondary hypogammaglobinemia likely related to Rituximab use. Since IVIG is an ancillary therapy for BK virus infection, this medication could be a confounding factor in our study. However, IVIG might have a small role in BK virus prevention since it has not proven to be more efficacious than immunosuppressive therapy reduction in renal transplant patients [23, 24].

This study has limitations that can limit its generalizability to all children with SLE. Our cohort sample size was small and adherence with scheduled visits affected data collection. The patients at our academic institution may have more severe SLE disease, requiring more intensive therapy. We included some patients with SLE who also met criteria for MCTD and Overlap syndrome and therefore required medications/biologics not typically used to treat SLE. As a result, treatment variability within the cohort may impact the overall immunosuppression level of each individual patient. Additionally, there is potential for exposure misclassification for medications that were self-reported during visits. Since we have no reason to believe that the classification would be biased in favor of one direction over another, we would call this non-differential misclassification. This would bias the results toward showing no difference [25]. Lastly, there is great heterogeneity amongst studies, as cited before, defining what a positive BK viral load is and what is a significant value to be concerned about. In the renal transplant literature, a cut off of >1×10^4^ BKV copies/ml is the threshold for possible BKV nephropathy [26]. However, this cut off is of uncertain value and may be too arbitrary for BKV nephropathy in non-transplant patients. By having various cutoff as positive BK viruria or viremia, the literature may underrepresent the true prevalence and/or risk of BK virus infection in patients with SLE. Thus, we decided that the presence of viruria or viremia, perhaps even at lower viral copies, in an immunocompromised patient should at least be explored in order to bring awareness to the physician about their patient’s immunosuppression status.

At this moment, it is difficult to ascertain to what degree of cellular, humoral or cytokine impairment leads to activation of BK virus since multiple immune pathways were affected simultaneously. It is possible that the BK virus prevalence may increase over time if this cohort were to be followed for a longer period of time.

## Conclusions

In summary, we observed transient BK virus reactivation in children with SLE. We were unable to identify any patient, laboratory, or medication factors linked to a higher risk for BK virus reactivation other than hydroxychloroquine. However, we did see that those with SLE in the context of MCTD and/or overlap syndrome had higher rates for BK virus infection. Thus, ongoing research is still warranted in a larger population to understand the impact of immunosuppression in children with SLE and risk for BK virus infections.

## Abbreviations

ANA: Antinuclear antibody
BKV: BK virus
HIV: Human immunodeficiency virus
IVIG: Intravenous immunoglobulin
MCTD: Mixed connective tissue disease
MMF: Mycophenolate mofetil
PCR: Polymerase chain reaction
RT-PCR: Real-time polymerase chain reaction
RTX: Rituximab
SLE: Systemic lupus erythematosus
VP: Viral capsid proteins
